# Influence of Extraction Methods on the Physicochemical and Biological Properties of Amazonian Beetle Larva Oil (*Speciomerus ruficornis*)

**DOI:** 10.1021/acsomega.5c11230

**Published:** 2026-06-12

**Authors:** Gabriel Araújo de Jesus, Allan Luiz Galvão Dickson, Vanessa Albuquerque de Mescouto, Maurílio Cunha Silva, Carmelita de Fátima Amaral Ribeiro, Carlos Emmerson Ferreira da Costa, Rodrigo Juliano Oliveira, Bruno Rafael Ribeiro de Almeida, Adauto Lima Cardoso, Renata Coelho Rodrigues Noronha, Luís Adriano Santos do Nascimento

**Affiliations:** † Amazon Oil Laboratory, Institute of Biological Sciences, 37871 Federal University of Pará (UFPA), Belém 66075-110, Brazil; ‡ Genetics and Cellular Biology Laboratory, Center for Advanced Studies of Biodiversity, Institute of Biological Sciences, Federal University of Pará (UFPA), Belém 66075-110, Brazil; § Food Science and Technology Laboratory, 306972 State University of Pará (UEPA), Salvaterra Campus, Salvaterra 68860-000, Brazil; ∥ Center for Studies in Stem Cells, Cell Therapy and Toxicological Genetics, School of Medicine, Federal University of Mato Grosso do Sul (UFMS), Campo Grande 79070-900, Brazil; ⊥ Department of Natural Sciences, State University of Pará (UEPA), Campus XVIII, Cametá 68400-000, Brazil

## Abstract

Background and aim: The Amazonian fruit *Astrocaryum vulgare* (Tucumã-do-Pará) plays a central economic and cultural role on Marajó Island, where a traditional oil known as “*óleo de bicho*” (larva oil), derived from *Speciomerus ruficornis* larvae within the seed, is widely used in food, medicine, and cosmetics. This study compared three extraction techniques [traditional frying, mechanical pressing, and ultrasound-assisted extraction (UAE)] in terms of oil yield, physicochemical integrity, and biological effects. Experimental procedure: Larva oil was extracted by frying (180 °C), mechanical pressing, and UAE (∼50 °C) and evaluated for yield, acidity, peroxide value, and fatty acid composition. Because the raw material showed signs of prior degradation, the study also assessed the robustness of each extraction method under compromised conditions. Biological assays included MTT-based cytotoxicity and RT-qPCR analysis of p16 and p21 expression in HaCaT and B16F10 cells, with statistical analysis performed by analysis of variance/Tukey (*p* < 0.05). Results and discussion: Frying produced the highest yield (45%), while UAE yielded oil with lower acidity (8.36 mg KOH/g) and peroxide value (3.97 mequiv O_2_/kg), and a balanced fatty acid profile (lauric, myristic, palmitic, and oleic acids). Cytotoxicity occurred only at the highest concentration tested (1777.35 μg/mL). Pressed oil altered p16 and p21 expression in a cell-type–dependent manner, whereas oil obtained by UAE had no significant effect on these genes, indicating no detectable cytotoxic or gene modulation effects under the tested conditions. Conclusion and implications: UAE showed the greatest preservation of the oil’s physicochemical integrity and molecular stability among the evaluated methods.

## Graphical abstract

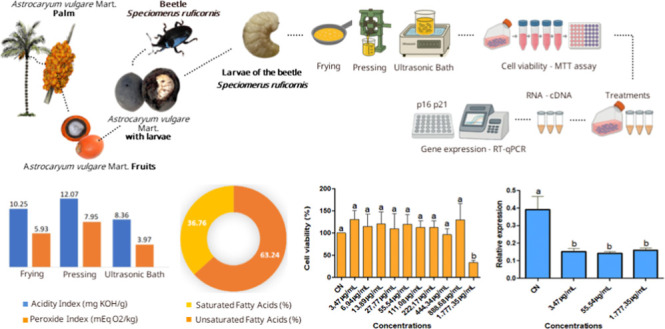

## 1 Introduction

The Amazon region has a rich biodiversity, including fruits such as açaí (*Euterpe oleracea*), bacaba (*Oenocarpus bacaba*), buriti (*Mauritia flexuosa*), and tucumã-do-Pará (*Astrocaryum vulgare* Mart.), whose pulps, peels, and seeds offer proteins, carbohydrates, fibers, lipids, vitamins, and minerals, as well as various bioactive compounds with high antioxidant activity.^1,2^ The region’s vegetable oils, including pracaxi oil (*Pentaclethra macroloba*), andiroba oil (*Carapa guianensis*), copaiba oil (*Copaifera*spp.), and buriti oil (*M. flexuosa*), have unique compositions and are traditionally used in medicinal, cosmetic, nutraceutical, and food products.^3^ Among these, tucumã-do-Pará stands out as a typical Amazonian palm with high potential for food, cosmetics, and oil production and is common on Marajó Island.

In addition to consuming the tucumã fruit in the form of juice or fresh pulp, local residents use bicho oil, obtained from the larva of a beetle of the Chrysomelidae family that lays an egg inside the seed; inside, the larva feeds on the seed endosperm. An entomological study was carried out in the municipality of Salvaterra (0°45′10″S 48°31′1″W), on Marajó Island, within a radius of approximately 20 km from the collection region of this work. The present work performed the taxonomic identification of these insects and concluded that the specimens were predominantly identified as *Speciomerus ruficornis* Germar.^4^ While this species is predominantly identified in the region, the taxonomic identification in our study was based on morphological traits and regional occurrence, rather than molecular confirmation, which represents a limitation of this study. This oil is one of the drivers of the economy in the communities of the municipality of Soure, on Marajó Island.^5^ The consumption of this larva oil is common in the region, and it is frequently associated with therapeutic benefits, including wound healing, anti-inflammatory, and analgesic effects, as well as uses for insomnia and even purported anticancer activity, although such claims remain anecdotal and scientifically unproven.^6^

The oil comes from larvae predominantly identified as *S. ruficornis*. The female beetle lays eggs on the seed surface, and upon hatching, the larvae enter the seed to feed on the almond. Despite the possibility that multiple eggs are laid in a single seed, typically only one larva survives due to resource competition or natural mortality.^7^ During development, the larva consumes the seed’s internal tissues until reaching the final stage, when it would normally pupate and emerge as an adult beetle. However, larvae are harvested for oil extraction prior to pupation.^6^ This developmental cycle is illustrated in Figure 1.

**Figure 1:**
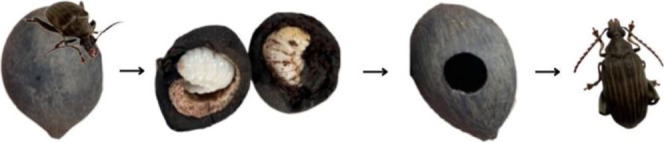
Illustration of the development stages of the beetle *Speciomerus ruficornis* Germar.

Traditionally, larva oil is extracted by frying, a common process among the Marajoara people and the only method documented in the literature.^6,8^ Frying, although simple, requires strict temperature control, since high temperatures accelerate the oil oxidation, while lower temperatures prolong the frying time and better preserve its physicochemical properties.^9,10^ Even with these precautions, the frying process does not appear to significantly compromise the traditional uses of larva oil, which is widely applied in cosmetic and medicinal contexts, according to local knowledge. Despite its widespread empirical use, such effects remain largely anecdotal and scientifically unvalidated, highlighting the need for robust experimental studies to assess the bioactivity and safety of Amazonian oil.

In vitro cell culture models are widely used to assess the cytocompatibility and potential toxicity of bioactive oils before their use in cosmetic or pharmaceutical formulations. Research indicates that cytotoxicity can be efficiently assessed using in vitro assays, providing preliminary safety data for further in vivo testing.^11^ This approach also enables rapid detection of potential adverse effects on human cells, reducing risks in early product development.^12^ The protective effects of an extract on cells are an important indicator of efficacy, especially in skin care applications.^13^

This work investigates the influence of different extraction methods on the chemical composition and biological properties of larva oil. Traditional frying was compared with mechanical pressing and ultrasound-assisted extraction (UAE), both conducted at lower temperatures to maintain the oil’s chemical stability. We assess how each extraction technique affects physicochemical parameters, fatty acid composition, cell viability, and p16/p21 gene expression in human HaCaT (human keratinocyte cell line) and murine B16F10 (murine melanoma cell line), providing insight into the therapeutic and toxicological implications of this Amazonian oil.

Despite the recognized ethnopharmacological use of this oil, no previous study has compared different extraction methods applied to an animal-derived larva oil under controlled laboratory conditions while simultaneously evaluating its biological impact at the molecular level. Given that lipid oxidation and fatty acid composition may influence oxidative stress- and senescence-related pathways, the cell cycle regulators p16 (CDKN2A) and p21 (CDKN1A) were selected as molecular markers to assess potential lipid-mediated cellular responses. Nevertheless, how extraction parameters influence not only the physicochemical quality but also the biological safety of larva oil remains poorly understood. Therefore, this study bridges a critical gap between traditional knowledge and experimental validation.

## 2 Materials and Methods

### 2.1 Sample Collection

Initially, surveys were conducted with residents of Soure (Marajó Island) to identify areas with high incidence of beetle larvae. Two communities actively involved in extraction activities were identified: Pedral (0°40′05.1″S 48°31′05.9″W) and Caju-una (0°37′47.2″S 48°29′13.2″W). After harvesting the tucumã seeds, they were broken on site with a knife and machete. A total of 1000 g of larvae were collected, stored in a Styrofoam cooler, and transported to the Food Technology Laboratory at UEPA (Salvaterra Campus) on Marajó Island, where oil extraction was performed. The frying extraction was carried out immediately after collection, following the traditional method used by local extractivists, whereas larvae intended for mechanical pressing and UAE were stored under freezing conditions (≈ −18 °C) for approximately 2 weeks prior to extraction.

Larvae used in this study were predominantly identified as *S. ruficornis* Germar based on morphological characteristics and regional occurrence without molecular taxonomic confirmation.

### 2.2 Morphological Characterization of Larvae

A total of fifty-five larvae were measured using a 6-in. (150 mm) caliper for length, width, thickness, and curvature. Each larva was then weighed on an analytical balance, as shown in Figure 2.

**Figure 2:**
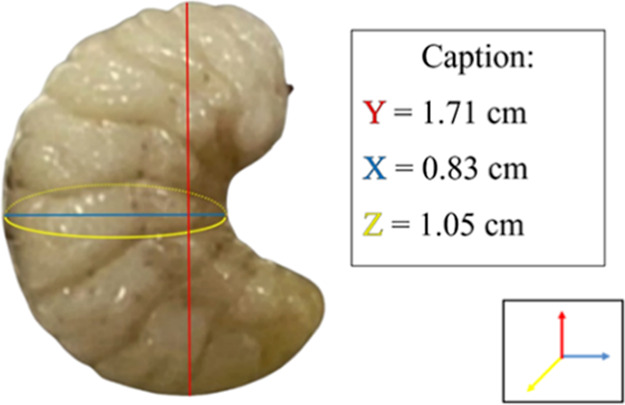
Morphometric measurement of *Speciomerus ruficornis* larva performed with a digital caliper. The red (*Y*), blue (*X*), and yellow (*Z*) axes represent the main dimensions used for size determination: length = 1.71 cm, width = 0.83 cm, and thickness = 1.05 cm.

### 2.3 Traditional Extraction of Larva Oil

The traditional method of obtaining larva oil is frying.^8,14^ Larvae were extracted from tucumã seeds by manually breaking the seeds and then frying them in aluminum containers on a stove at approximately 180 °C for 5 min, until moisture was visibly reduced. The contents were filtered using Whatman grade 40 filter paper (≈8 μm pore size, GE Healthcare, USA) to separate solids from the oil. The filtered oil was collected in amber-glass bottles, sealed, and stored at 4 °C for further analysis.

### 2.4 Extraction of Larva Oil by Mechanical Pressing

This procedure followed the methodology of.^15^ For oil extraction by pressing, the larvae were initially preheated in a drying and sterilization oven (SL-100, Solab, Brazil) at 130 °C for 30 min to reduce moisture content and facilitate oil release during pressing. After oven drying, the larvae were subjected to microwave pretreatment (60 W for 1 min) to promote cell disruption prior to mechanical pressing. This brief pretreatment was applied to assist oil mobilization; however, sample temperature was not directly monitored during this step, and localized heating of the lipid phase cannot be excluded. After heat treatment, the oil was extracted using a manual bench press under continuous mechanical pressure.

### 2.5 Extraction of Larva Oil by UAE

UAE was adapted from ref 16 and performed using a Solid Steel 6 L ultrasonic bath (40 kHz, 160 W, fixed bath temperature of 35 °C). To maintain the internal extraction temperature around 50 °C, preheated water (≈60 °C) was added to the bath prior to each run, while the bath temperature was set at 35 °C. Initially, the larvae were preheated in an oven at 60 °C for 30 min to facilitate oil release. After preheating, the larvae were macerated in a porcelain mortar with a 100 mL pestle, weighed, mixed with distilled water in a 6:1 ratio (50 g of macerated larvae to 300 mL of water), and placed in the ultrasonic bath. The extraction process was conducted under optimized conditions: ultrasonic power of 160 W, effective extraction temperature of 50 °C, extraction time of 50 min, and ultrasonic interval of 2 s. After extraction, the suspended oil was collected with a pipet and centrifuged in a bench centrifuge (Quimis Q222T, Diadema, Brazil) at 4000 rpm (≈2200 × g) for 10 min to remove solid residues. The upper lipid fraction was carefully collected for subsequent analysis.

### 2.6 Total Yield of Extracted Oils

The total oil yield obtained by the different extraction methods was determined based on the methodology of ref 14 and calculated by dividing the mass of extracted oil (g) (*W*_oil_) by the total mass (*g*) of larvae (*W*_larvae_), as shown in eq 1.

Equation 1 shows the calculation of the total yield of extracted larva oil.

$$Oil\;yield\;\left(\%\right)=\frac{W_{oil}}{W_{larvae}}\times 100$$ 1

### 2.7 Oil Stability Study

Analyses of physicochemical indices were performed according to the American Oil Chemists’ Society (AOCS) Official Methods and Recommended Practices, Sixth Edition (2009).^17^ The specific methods utilized were: density (AOCS *Cc* 10c-95, reapproved 2003), acid number/free fatty acids (AOCS Ca 5a-40, reapproved 2009), peroxide value (AOCS Cd 8b-90, reapproved 2017), saponification number (AOCS Cd 3-25, reapproved 1997 and revised 2001–2002), and fatty acid composition (AOCS Ce 2-66, reapproved 1997). All physicochemical analyses for each extraction method were performed in triplicate (*n* = 3).

### 2.8 Characterization of Fatty Acids

The fatty acid composition present in the oil was determined by gas chromatography (GC). The analysis was performed in a Shimadzu GC-2010 gas chromatograph equipped with a flame ionization detector, with the following specifications: TG-WAX MS capillary column that is 30 m in length, 0.32 mm in internal diameter, and 0.25 μm (micrometer) in film thickness. Helium gas was used as the carrier gas at a rate of 1.0 mL/min (milliliter/min). The injection volume was 1.0 μL. The oven temperature program was as follows: initial temperature of 80 °C held for 2 min, increased to 180 °C at a rate of 10 °C/min, then increased to 250 °C at 10 °C/min, and held for 5 min. Fatty acids were converted to methyl esters following the AOCS Ce 2-66 method (Revised 2009), using BF_3_-methanol (Sigma-Aldrich) as the methylating agent. The resulting methyl esters were extracted with heptane, separated with saturated sodium chloride solution, and subsequently analyzed by GC. The fatty acid profile for each extraction method was determined in triplicate (*n* = 3).

### 2.9 Cell Culture

The HaCaT (human keratinocytes) cell line from CLS Cell Lines Service (Eppelheim, Germany) and B16F10 (murine melanoma) cell line from the American Type Culture Collection (ATCC, Manassas, VA, USA) were selected for this study. The cells were seeded in 25 cm^2^ cell culture flasks with a filter containing 3 mL of Dulbecco’s Modified Eagle’s Medium (DMEM) (high-glucose) medium supplemented with 10% fetal bovine serum and 1% Penicillin–Streptomycin (all from Gibco, Thermo Fisher Scientific, USA) and maintained in an incubator at 37 °C and 5% CO_2_ until 90–100% confluence. The cells were trypsinized (Trypsin–EDTA, Gibco) and transferred to new flasks every 3–5 days.

### 2.10 Cell Viability Assessment: MTT Assay

Cell viability was evaluated using the MTT assay (3-(4,5-dimethylthiazol-2-yl)-2,5-diphenyl tetrazolium bromide). HaCaT (human keratinocyte) and B16F10 (murine melanoma) cells were seeded at 2 × 10^5^ cells per well in 96-well plates containing 100 μL of DMEM and incubated for 24 h at 37 °C and 5% CO_2_. Stock solutions of each oil were prepared in dimethyl sulfoxide (DMSO), an organic solvent capable of solubilizing lipophilic compounds and improving their dispersion in aqueous media. These solutions were then diluted in DMEM to obtain the desired test concentrations, ensuring that the final DMSO concentration, including all treated and control wells, did not exceed 1% (v/v). All working solutions were freshly prepared and vortexed prior to addition to the cells to ensure homogeneous dispersion, and no visible phase separation was observed during the incubation period. For validation purposes, an additional solvent control (1% DMSO in DMEM) was included to confirm the absence of vehicle cytotoxicity; this control was not used in comparative statistical analyses. After an additional 24 h, the medium was removed, and 100 μL of MTT solution (Sigma-Aldrich, USA) (0.5 mg/mL in medium) was added to each well for 4 h. Following incubation, the MTT solution was discarded, and 100 μL of dimethyl sulfoxide (DMSO) (Sigma-Aldrich, USA) was added to dissolve the formazan crystals. Absorbance was measured at 570 nm using a BioTek Epoch microplate spectrophotometer (Agilent Technologies, USA).^18^ Absorbance values were normalized to the untreated control, which was set at 100%. Each treatment was performed in five technical replicates and three independent biological replicates. Ten concentrations of oil (3.47, 6.94, 13.89, 27.77, 55.54, 111.08, 222.17, 444.34, 888.68, and 1777.35 μg/mL) were evaluated. The negative control (NC) was medium with 1% DMSO (Dinâmica, Brazil). Data were analyzed by analysis of variance (ANOVA), followed by Tukey’s test (GraphPad Prism 5, GraphPad Software, USA), with significance at *p* < 0.05.

### 2.11 RT-qPCR

HaCaT and B16F10 cells were treated for 12 h with larva oil extracted by mechanical pressing or UAE. Three concentrations were selected: 3.47 μg/mL, 55.54 μg/mL, and 1777.35 μg/mL. Total RNA was isolated using a PureLink RNA Mini Kit (Thermo Fisher Scientific, USA), and RNA quality was checked on a NanoVue spectrophotometer (Cytiva, USA). RNA was reverse-transcribed with the High-Capacity cDNA Reverse Transcription Kit (Thermo Fisher Scientific, USA). PCR primers were synthesized commercially (Exxtend, Brazil) based on sequences from the literature:^19,20^ p16 (Forward: CAACGCACCGAATAGTTACGG; Reverse: GCGCAGTTGGGCTCCG) and p21 (Forward: TGATGCGCTAATGGCGGGCT; Reverse: TGCTGGTCTGCCGCCGTTTT). cDNA (4 ng/μL) was amplified with Real Q Plus 2X Master Mix (Ampliqon) and 400 nM primers in 20 μL reactions. Cycling conditions were 95 °C for 10 min; 40 cycles of 95 °C for 15 s and 60 °C for 1 min. Amplification was detected with a Bio-Rad CFX Maestro system (Bio-Rad Laboratories, USA). A dissociation curve confirmed specificity. Data were normalized using *YWHAZ* (Forward: CCGTTACTTGGCTGAGGTTG; Reverse: TGCTTGTTGTGACTGATCGAC) as a reference gene.^21^ All RT-qPCR reactions were performed in duplicate (two technical replicates) for each of three independent biological replicates. ΔΔ*C*q values were calculated by the comparative method.^22^

### 2.12 Statistical Analysis

The results of the physicochemical characterization were expressed as the mean ± standard deviation (SD) of three replicates. These data were subjected to a one-way ANOVA, and the means were compared using Tukey’s posthoc test. For the cell-based assays, data from cell viability and gene expression were also expressed as mean ± SD, and statistical analyses were performed as described in their respective sections. All statistical analyses were performed using GraphPad Prism 5 (GraphPad Software Inc., San Diego, CA, USA), and differences were considered statistically significant when *p* < 0.05.

## 3 Results

### 3.1 Morphometric Characterization of Larvae

Measurements of 55 larval specimens showed heterogeneous sizes: weights ranged from 1.14 to 1.88 g, lengths from 1.24 to 1.96 cm, widths from 0.68 to 0.98 cm, and thicknesses from 0.79 to 1.31 cm.^23^ Table 1 summarizes these morphometric data.

**Table 1:** Morphometric Analysis of Amazonian Beetle Larvae (*n* = 55) Performed with a Caliper^a^

weight (g)	length (cm)	width (cm)	thickness (cm)
1.51 ± 0.37	1.71 ± 0.26	0.83 ± 0.15	1.05 ± 0.26

a Data are presented as mean ± standard deviation.

### 3.2 Larva Oil Extraction by Different Methods

From the 1000 g of collected larvae, three 300 g subgroups were prepared for extraction trials (frying, pressing, and UAE), leaving a surplus of 100 g.

After extraction, physicochemical analyses were performed on the oils obtained to evaluate possible changes in their quality and fatty acid compositions. The oil yields differed among methods: frying yielded 45% oil, pressing yielded 40%, and UAE yielded 26% (Table 2).

**Table 2:** Physicochemical Properties of Fried, Press-Extracted, and Ultrasound-Assisted Extracted Larva Oils^a^ ^,^ ^b^

property	fried	press	UAE
yield (%)	45 ± 0.01^a^	40 ± 0.01^b^	26 ± 0.02^c^
acidity index (mg KOH/g)	10.25 ± 0.00^b^	12.07 ± 0.09^a^	8.36 ± 0.01^c^
peroxide index (mEq O_2_/kg)	5.93 ± 0.01^b^	7.95 ± 0.01^a^	3.97 ± 0.01^c^
saponification index (mg KOH/g)	227.72 ± 5.82^a^	234.43 ± 4.33^a^	238.13 ± 3.17^a^
density at 20 °C (g/mL)	0.91 ± 0.00^a^	0.91 ± 0.00^a^	0.91 ± 0.00^a^

a Different superscript letters (a, b, c) in the same row indicate a significant difference between the extraction methods according to the Tukey test (*p* < 0.05).

b Data are presented as mean ± standard deviation (*n* = 3).

Physicochemical analyses showed that oil obtained by UAE exhibited the lowest degradation indices, reflecting better preservation of physicochemical integrity under the tested conditions, with an acidity of 8.36 mg of KOH/g and a peroxide value of 3.97 mEq of O_2_/kg. The elevated acidity levels in all oil samples are likely a consequence of larval degradation during transport and storage before the extraction. This context is an important consideration when comparing our findings with those of other studies performed on freshly collected larvae. The frying method produced slightly higher acidity (10.25 mg of KOH/g and 5.93 mEq of O_2_/kg) for peroxide values, indicating a decline in quality. Pressing resulted in the highest acidity (12.07 mg of KOH/g and 7.95 mEq of O_2_/kg) for peroxide values, indicating comparatively lower quality among the tested methods.

The quality of the oil was evaluated in comparison with the standards defined by Codex,^24^ revealing that all samples exceeded the recommended limits for acidity and peroxide values, highlighting the impact of storage and handling on oil integrity.

It is worth highlighting that due to the animal origin and limited availability of the oil, subsequent biological assays focused on oils obtained by mechanical pressing and UAE, as these methods offer greater process control and scalability.

### 3.3 Comparison of the Chromatographic Composition of Larva Oil Extracted by Different Methodologies

Fatty acid concentrations were determined by chromatography (Table 3). The fatty acid profiles were virtually identical across extraction methods, with similar proportions of lauric, myristic, palmitic, and oleic acids, indicating that the core lipid composition was preserved, despite differences in quality parameters.

**Table 3:** Fatty Acid Concentration in Oils Extracted by Different Methodologies

fatty acids	concentration (%)		
(*n* ^0^ of carbons/*n* ^0^ of double bonds)	fried	press	UAE
capric (C10:0)	0.11 ± 0.01	0.14 ± 0.02	0.12 ± 0.01
lauric (C12:0)	22.60 ± 0.45	23.68 ± 0.58	23.87 ± 0.51
myristic (C14:0)	18.46 ± 0.07	18.61 ± 0.05	18.52 ± 0.07
pentadecanoic (C15:0)	0.02 ± 0.00	0.02 ± 0.00	0.02 ± 0.00
palmitic (C16:0)	17.12 ± 0.28	16.91 ± 0.17	16.56 ± 0.21
palmitoleic (C16:1)	0.33 ± 0.02	0.30 ± 0.02	0.28 ± 0.02
margaric (C17:0)	0.06 ± 0.01	0.05 ± 0.01	0.03 ± 0.01
stearic (C18:0)	4.22 ± 0.00	4.22 ± 0.01	4.01 ± 0.02
oleic (C18:1)	32.58 ± 0.39	31.79 ± 0.40	32.08 ± 0.39
linoleic (C18:2)	4.24 ± 0.08	3.93 ± 0.09	3.97 ± 0.07
araquidic (C20:0)	0.13 ± 0.01	0.13 ± 0.00	0.12 ± 0.01

### 3.4 Cell Viability Assay (MTT) in Human Keratinocyte Cells (HaCaT)

In the HaCaT keratinocyte cell line, press-extracted oil did not significantly affect cell viability at 24 or 48 h, except at the highest concentration (1777.35 μg/mL), which induced cell death (Figure 3A,B). Oil obtained by UAE showed a similar pattern, with no significant effect at any concentration except 1777.35 μg/mL (Figure 3C,D). Under the tested conditions, neither oil reduced keratinocyte viability below 1000 μg/mL.

**Figure 3:**
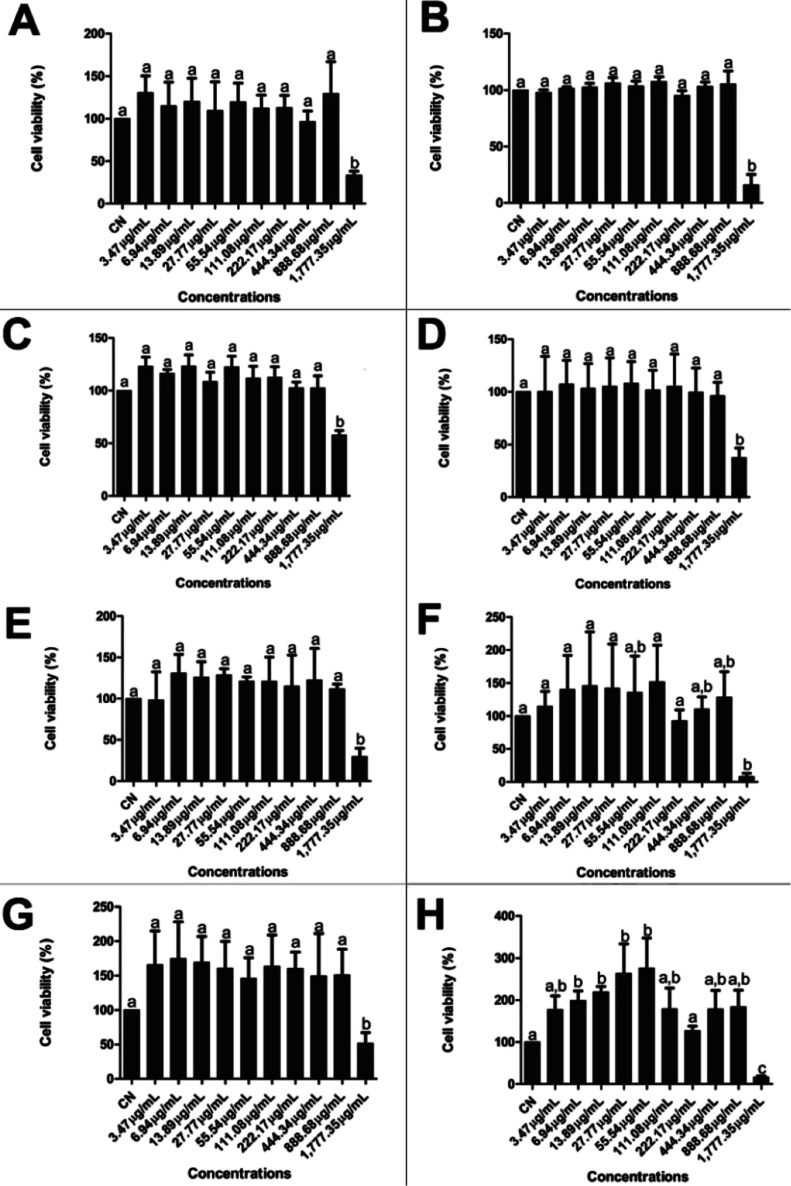
Cell viability (mean ± SD) of HaCaT and B16F10 cell lines treated with press-extracted larva oil or ultrasound-assisted extracted larva oil at different concentrations for 24 and 48 h, evaluated by the MTT assay. Panels (A) and (B) show HaCaT cells treated with press-extracted larva oil for 24 and 48 h, respectively; (C) and (D) show HaCaT cells treated with ultrasound-assisted extracted larva oil for 24 and 48 h, respectively; (E) and (F) show B16F10 cells treated with press-extracted larva oil for 24 and 48 h, respectively; and (G) and (H) show B16F10 cells treated with ultrasound-assisted extracted larva oil for 24 and 48 h, respectively. Statistical comparisons between treatments and the NC (0 μg/mL) were performed using one-way ANOVA, followed by Tukey’s post hoc test (*p* < 0.05).

### 3.5 Cell Viability (MTT Assay) in Murine Melanoma Cells (B16F10)

In the B16F10 melanoma cell line, press-extracted oil affected cell viability only at the highest concentration (1777.35 μg/mL) after both 24 and 48 h of exposure, with no significant differences observed at lower concentrations (3.47–888.68 μg/mL) (Figure 3E,F).

Oil obtained by UAE produced a similar pattern: after 24 h, viability decreased only at 1777.35 μg/mL (Figure 3G). However, after 48 h, small but significant increases were detected at intermediate concentrations (6.94–55.54 μg/mL) compared with the control, followed by a marked reduction at 1777.35 μg/mL (Figure 3H). This biphasic response is consistent with a hormetic pattern commonly observed in dose–response studies.

### 3.6 Gene Expression of p16 and p21 in HaCaT and B16F10 Cell Lines Treated with Press-Extracted Larva Oil

To evaluate the expression of the p16 (CDKN2A) and p21 (CDKN1A) genes, HaCaT and B16F10 cells were exposed for 12 h to three concentrations of press-extracted larva oil (3.47, 55.54, and 1777.35 μg/mL).

In HaCaT cells, p16 expression increased significantly only at 1777.35 μg/mL, while lower concentrations showed no change relative to the control (Figure 4A). In contrast, B16F10 cells exhibited a significant reduction in p16 expression at all concentrations tested (3.47–1777.35 μg/mL) (Figure 4E).

**Figure 4:**
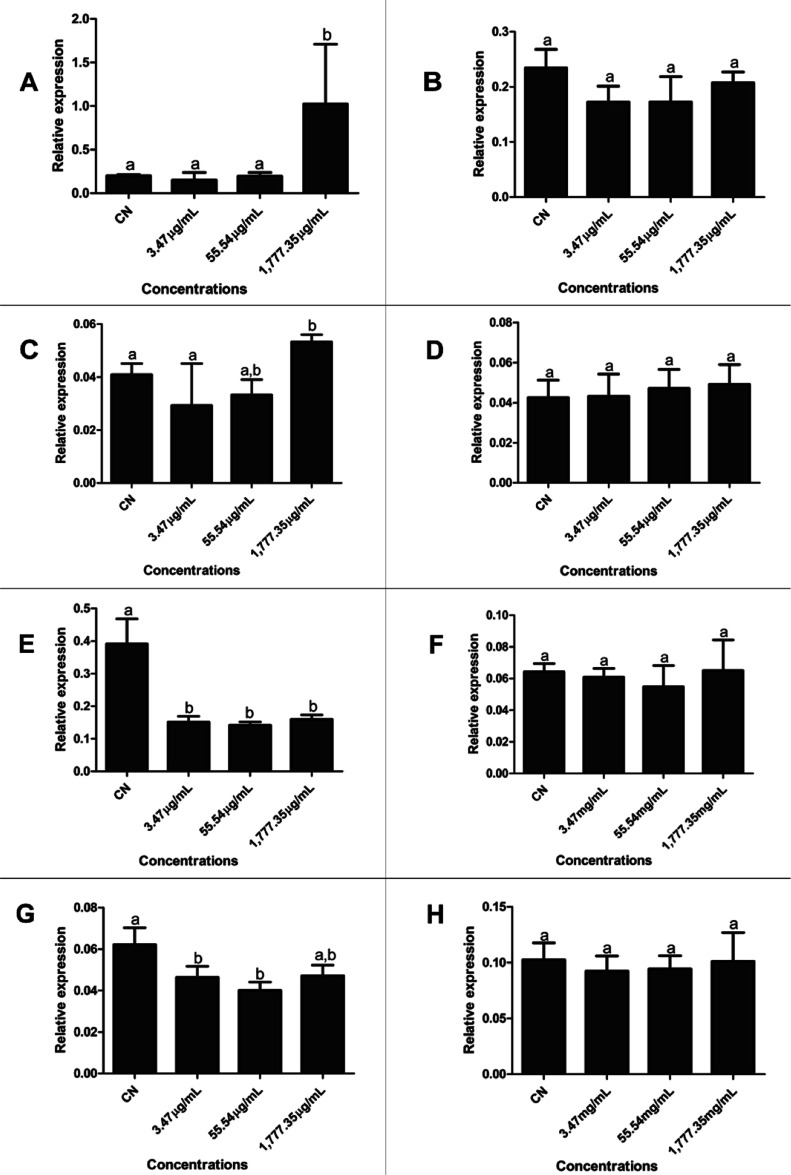
Relative expression levels of the p16 gene in HaCaT (A) and B16F10 (E) cell lines treated with larva oil obtained by the pressing method, analyzed by RT-qPCR and normalized to the YWHAZ control. Expression levels of the p21 gene in HaCaT (C) and B16F10 (G) cell lines treated with larva oil obtained by the pressing method, analyzed by RT-qPCR and normalized to the YWHAZ control. Expression levels of the p16 gene in HaCaT (B) and B16F10 (F) cell lines treated with larva oil obtained by UAE, analyzed by RT-qPCR and normalized to the YWHAZ control. Expression levels of the p21 gene in HaCaT (D) and B16F10 (H) cell lines treated with larva oil obtained by UAE, analyzed by RT-qPCR and normalized to the YWHAZ control. The comparison between treatments and the NC (0 μg/mL) was performed using ANOVA, followed by Tukey’s test.

Regarding p21 expression, no significant change was observed in HaCaT cells at any concentration (Figure 4C). However, in B16F10 cells, p21 expression decreased at 3.47 μg/mL and 55.54 μg/mL but remained unaltered at 1777.35 μg/mL (Figure 4G).

These results suggest that the modulation of cell cycle regulators by the larva oil depends on the cell type and concentration. The p16 upregulation in HaCaT cells at high doses may indicate a senescence-related response, whereas the downregulation of p16 and p21 in melanoma cells could reflect disruption of tumor suppressor pathways involved in cell cycle control.^25–28^

### 3.7 Gene Expression of p16 and p21 in HaCaT and B16F10 Cell Lines Treated with Larva Oil Extracted by UAE

HaCaT and B16F10 cells treated for 12 h with oil obtained by UAE (3.47, 55.54, and 1777.35 μg/mL) showed no statistically significant changes in p16 or p21 expression at any concentration (Figure 4B, D, F, H). In both cell lines, p16 and p21 levels remained comparable to the control across all tested doses. These findings indicate that oil obtained by UAE did not induce detectable modulation of p16 or p21 expression under the tested conditions.

## 4 Discussion

The present work expands upon previous analyses of Amazonian larva oil, including assessments of acidity, unsaponifiable matter, iodine index, saponification, and chromatographic profile,^6,29^ by incorporating new physicochemical analyses and, for the first time, evaluating its cytotoxicity and gene modulation potential in human and murine cell models.

Two extraction techniques were applied, following the methodologies described by ref 15 (pressing) and ref 16 (UAE), in addition to the traditional frying method, to evaluate how these approaches influence oil yield, quality, and stability.

The first key finding of this study is that all extracted oils showed elevated acidity and peroxide levels (Table 2), exceeding the standards for edible oils.^24^ This suggests that the larval raw material may have undergone degradation during transport and storage prior to extraction. While this represents a limitation in terms of final product quality, it also provides an opportunity to assess the robustness of each extraction method under realistic stress conditions. The central question therefore shifts from “which method produces the best oil?” to “which method best mitigates further degradation of a compromised raw material?”.

Further context for these results involves the biological source itself. A study conducted by Silva et al.^4^ on Marajó Island supports the assumption that the larvae analyzed in the present study were predominantly of the species *S. ruficornis*. However, no formal taxonomic identification of the collected larvae was performed, which represents an important limitation. The potential presence of different chrysomelid species may have contributed to the variability observed in oil yield and composition. Therefore, future studies should incorporate taxonomic identification protocols prior to extraction to ensure greater reproducibility and accuracy in the characterization of Amazonian larva oil.

The direct impact of these raw material conditions becomes evident when contrasting our results with those from the literature. In previous studies, such as ref 14, oil extracted from freshly collected larvae using the frying method exhibited low acidity (1.5 ± 0.3 mg KOH/g) and peroxide values (3.5 ± 0.8 mequiv O_2_/kg), remaining within the acceptable limits established by.^24^ In contrast, in the present study, oils extracted by the same method exhibited significantly higher acidity (10.24 ± 0.0 mg KOH/g) and peroxide values (5.93 ± 0.01 mequiv O_2_/kg), indicating reduced oil quality. These findings highlight the importance of optimizing postharvest handling and minimizing the time between larval collection and processing, particularly under tropical conditions, where high humidity and temperature can accelerate oil degradation. Microbial activity from bacteria and fungi present on or inside the larvae may rapidly increase if the material is not refrigerated or processed quickly, promoting the breakdown of lipids and raising the oil’s acidity.^30^ Additionally, enzymes can hydrolyze triglycerides into free fatty acids and glycerol, further raising acidity levels.^31^ These mechanisms help explain the high acidity observed and underscore the need for prompt processing and proper storage to maintain oil quality.

Furthermore, extraction parameters, particularly temperature and oxygen exposure, were critical for maintaining the oil quality. Oils processed at higher temperatures or subjected to greater mechanical stress exhibited elevated acidity and oxidative indices, likely resulting from intensified lipid oxidation and triglyceride hydrolysis into free fatty acids during both preprocessing storage and thermal treatment.^32^ Conversely, UAE, performed at lower temperatures and reduced oxygen exposure, preserved the oil integrity more effectively. Despite all oils exceeding the recommended acidity limit of 4.0 mg KOH/g,^24^ peroxide values remained below the critical threshold of 15 mequiv O_2_/kg, indicating that oxidative degradation was still in its early stages.

Regarding extraction methods, frying yielded the highest oil recovery (45%), representing a practical option for community use. Pressing, although performed at lower nominal temperatures and following the method described by ref 15, resulted in oils with the highest acidity (12.07 ± 0.09 mg KOH/g) and peroxide values (7.95 ± 0.01 mequiv O_2_/kg). This lower quality may be associated with greater exposure to environmental conditions during processing and to the use of a manual press not specifically designed for high-purity oil extraction, factors that can promote oxidation and hydrolysis reactions. In addition, a brief microwave pretreatment was applied prior to mechanical pressing to promote cell disruption and facilitate oil release; this pretreatment was conducted at moderate power for a short duration, but the sample temperature was not directly monitored, and localized heating of the lipid phase cannot be excluded. Consequently, this method requires further refinement and optimization, particularly regarding pretreatment conditions, to minimize potential thermal and oxidative degradation pathways. In contrast, UAE, adapted from ref 16, yielded oil with the greatest preservation of physicochemical integrity, presenting lower acidity (8.36 ± 0.01 mg KOH/g) and peroxide values (3.97 ± 0.01 mequiv O_2_/kg) compared to the other methods. Its lower operating temperature (∼50 °C) likely contributed to minimizing oxidative reactions and preserving both physicochemical and organoleptic properties.

It should be noted that, although the overall quality of the starting material was a limiting variable, as indicated by the high acidity values across all samples, the relative differences between the methods remain valid. The fact that UAE achieved superior results even under these conditions supports the effectiveness of the method in preserving the oil’s chemical integrity.

Despite these differences in physicochemical quality, chromatographic analyses revealed no significant variation in the fatty acid composition among the three extraction methods. This confirms that the core lipid profile of larva oil remains chemically stable, regardless of the extraction technique.

In summary, frying remains the most viable method for local and community applications due to its practicality and higher yield. Pressing shows potential for industrial-scale production but requires oil refining to improve quality. UAE, although less accessible for routine use, yielded oils with improved chemical quality and may represent a suitable method for clinical applications and biotechnological research. However, as all oils exhibited acidity values above the 4.0 mg KOH/g limit established for edible oils,^24^ further evaluation is required before considering oral use. Topical and dermocosmetic applications may be explored following refinement and quality improvement. The superior quality reported in previous studies is likely linked to the immediate extraction of oil from freshly harvested larvae, a traditional practice of extractivists in the Marajó region, which appears to minimize degradation during storage and transport. Thus, the choice of extraction method should take into account not only production yield and operational cost, but also chemical stability, postharvest handling, and the intended end use, with particular emphasis on ensuring safety for topical and pharmaceutical applications.

Table 3 reveals that the oils extracted by different methodologies showed high concentrations of lauric acid (C12:0), myristic acid (C14:0), palmitic acid (C16:0), and oleic acid (C18:1). As recommended by nutritional guidelines, the intake of saturated fatty acids is important for health but should be moderate.^33^

Lauric acid, found in foods such as coconut oil and breast milk, has antimicrobial properties against viruses, bacteria, and fungi, as well as stimulating the immune system and being effective in combating acne.^34–36^ Myristic acid, found in foods of plant and animal origin, has demonstrated anti-inflammatory and antinociceptive effects, being associated with the nitrergic system in recent studies.^37^ Palmitic acid, present in meat, dairy products, and tropical oils, in addition to being naturally produced by the body, has anti-inflammatory activities and therapeutic potential against fungi under controlled conditions.^38^ As it is the most abundant fatty acid in human sebum, it is widely used in cosmetics to treat skin pathologies and restore the skin barrier.^39^

The prevalence of these saturated fatty acids suggests potential applications of larva oil in formulations for skin protection, topical antimicrobial delivery, and as adjuncts in wound healing therapies. It is important to note that this study focused exclusively on oil extracted from larvae, and no direct comparison was performed with oils obtained from nonparasitized tucumã seeds. Such comparisons would be valuable to clarify the metabolic contribution of the larva to the final oil composition and should be addressed in future studies.

The composition of these fatty acids varies depending on the extraction method, and their oils can offer benefits such as strengthening the skin barrier, antioxidant, anti-inflammatory, and antimicrobial properties, in addition to helping with wound healing and having reported–related activity in specific experimental models.^40^ However, it is important to consider the risks associated with excessive consumption of saturated fatty acids, such as heart disease and cancer, as warned by the WHO, which recommends limiting their intake to 5–6% of daily caloric intake.

Among the fatty acids analyzed, oleic acid (C18:1) had the highest concentration. This unsaturated fatty acid is known to modulate inflammation, improve wound healing, reduce bad cholesterol (low-density lipoprotein), and increase good cholesterol (high-density lipoprotein), in addition to protecting cells against oxidative damage.^41^ More recent studies indicate autophagy-dependent anticancer-related effects of oleic acid in specific in vitro models, such as human hepatocellular carcinoma cell lines, as well as antioxidant and anti-inflammatory properties.^42^

Its abundance in larva oil composition highlights its potential relevance for dermatological applications. The cell viability assay was performed by using oils extracted by mechanical pressing and UAE. The results showed that at the concentrations tested, neither oil significantly reduced cell viability, except at the highest concentration evaluated. These findings are consistent with the fatty acid composition of larva oil, which is rich in compounds that have been previously reported as compatible with skin cell models. Previous studies using oils of similar composition, such as black soldier fly larvae oil and fish oil, have demonstrated cell viability preservation associated with antifungal, antimicrobial, antioxidant, and skin barrier maintenance properties.^43,44^ In the context of larva oil, the presence of fatty acids known for their incorporation into products with pharmacological potential reinforces their relevance for further evaluation. Collectively, these findings suggest that larva oil may represent a promising candidate for cosmeceutical and dermatological applications; however, further studies are necessary to assess the formulation stability, skin permeation, and in vivo safety.

Unsaturated fatty acids, particularly polyunsaturated fatty acids (PUFAs), play key roles in cell viability by modulating membrane fluidity, intracellular signaling, and apoptosis.^45,46^ Oleic acid, present in larva oil, is associated with energy metabolism and cell survival in various cell models, including mesenchymal stem cells.^47^ In the present work, the composition of larva oil, enriched with such compounds, supports the absence of cytotoxicity observed in both tested cell lines.

Extraction methods directly influenced oil properties, as further summarized in Table 4.

**Table 4:** Integrative Summary of Oxidative Status, Dominant Fatty Acids, and Biological Responses of Larva Oil Obtained by Different Extraction Methods

extraction method	oxidative status^a^	dominant fatty acids	biological response (summary)	overall assessment
fried	elevated oxidative indices	C12:0, C14:0, C16:0, C18:1	not evaluated	suitable for traditional use
press	elevated oxidative indices	C12:0, C14:0, C16:0, C18:1	gene modulation at high dose	requires refinement
UAE	lower oxidative indices	C12:0, C14:0, C16:0, C18:1	no cytotoxicity or gene modulation	stable biological profile

a Based on the peroxide and acidity indices.

UAE exhibited a comparatively more stable profile with respect to cell viability and physicochemical parameters with lower oxidative indices and no detectable gene modulation under the tested conditions, supporting its use in studies involving cell-based and biotechnological applications.

At the highest concentration (1777.35 μg/mL), press-extracted oil increased p16 expression in HaCaT cells, consistent with a stress-associated transcriptional response. The p16 gene plays a central role in cell cycle regulation and is frequently upregulated in response to cellular stress signals.^25,48^ Conversely, in B16F10 melanoma cells, press-extracted oil induced a significant reduction in p16 expression at all tested concentrations. While definitive conclusions cannot be drawn without complementary functional assays, these results indicate cell-type-dependent differences in p16 transcriptional regulation.

Similarly, p21, another key regulator of cell cycle control and stress responses,^49,50^ was not significantly affected in HaCaT cells treated with press-extracted oil. However, in B16F10 cells, p21 expression decreased at 3.47 μg/mL and 55.54 μg/mL. In contrast, oil obtained by UAE did not significantly alter p16 or p21 expression in either cell line.

Studies have shown that exposure to lipids, including saturated and unsaturated fatty acids, can influence the expression of genes involved in cell cycle regulation, including p16 and p21.^26^ The absence of detectable modulation in cells treated with oil obtained by UAE may be associated with the lower oxidative indices observed for this sample, since p16 and p21 are known to respond to oxidative stress and DNA damage.^25,48–50^ In this context, the improved chemical stability of oil obtained by UAE may be associated with the lack of transcriptional alteration observed here.

The literature associates altered p16 and p21 expression with tumor progression and therapy resistance,^27,28,51^ reinforcing the importance of balanced regulation in cellular homeostasis. Taken together, the modulation patterns observed in this study, particularly the absence of alterations induced by oil obtained by UAE, indicate a more stable transcriptional profile. Although only mRNA levels were evaluated in the present study and protein-level confirmation was not performed, these findings support further mechanistic and in vivo studies to better define the biological behavior of larva oil.

## 5 Conclusions

This study demonstrated that the extraction method influences the physicochemical quality and biological behavior of *S. ruficornis* larval oil. Among the evaluated techniques, frying provided the highest yield, whereas UAE produced oil with lower acidity and peroxide values and a fatty acid composition dominated by lauric, myristic, palmitic, and oleic acids. No cytotoxicity was observed below 1000 μg/mL under the tested conditions, and gene expression analysis revealed that only the pressed oil modulated p16 and p21 expression in a cell type-dependent manner, including p16 downregulation in melanoma cells.

UAE yielded oil with greater chemical stability and no detectable cytotoxicity or gene expression modulation under the experimental conditions evaluated. The association between chemical integrity and molecular responses suggests that minimizing oxidative degradation may help preserve normal gene expression patterns. Therefore, UAE represents a promising approach for obtaining chemically stable larval oil with potential for topical and biotechnological applications. These findings contribute to the scientific understanding of Amazonian larval oil and its potential technological applications.
